# Ocular abnormalities in a large patient cohort with retinitis pigmentosa in Western China

**DOI:** 10.1186/s12886-020-01797-z

**Published:** 2021-01-18

**Authors:** Lian Tan, Yanling Long, Ziyang Li, Xi Ying, Jiayun Ren, Cheng Sun, Xiaohong Meng, Shiying Li

**Affiliations:** 1 Southwest Eye Hospital/Southwest Hospital, Third Military Medical University (Army Medical University), Chongqing, 400038 China; 2 Key Lab of Visual Damage and Regeneration & Restoration of Chongqing, Chongqing, 400038 China

**Keywords:** Retinitis pigmentosa, Ocular abnormalities, Macular abnormalities, Cataract, Glaucoma, Best corrected visual acuity

## Abstract

**Background:**

To report the prevalence of ocular abnormalities and investigate visual acuity in a large cohort of retinitis pigmentosa (RP) patients in Western China.

**Methods:**

The medical records and ophthalmic examination reports of 2127 eyes of 1065 RP patients at one eye hospital were retrospectively reviewed to determined the prevalence of ocular abnormalities and the relationship between best corrected visual acuity (BCVA) and macular abnormalities.

**Results:**

Nyctalopia (58.2%) and blurred vision (27.1%) were the leading reasons for RP patients to request an ophthalmic examination. BCVA measurements in the better eyes at first clinical presentation showed that 304 patients (28.5%) were categorised as blind and 220 patients (20.7%) as low vision. The most common ocular abnormalities were macular abnormalities (59.7%) and cataracts (43.1%). The macular abnormalities included epiretinal membranes (51.1%), cystoid macular edema (18.4%), vitreomacular traction syndrome (2.4%), macular holes (2.3%) and choroidal neovascular membranes (0.05%). Glaucoma was found in 35 eyes (1.6%). The proportions of epiretinal membranes (*p* = 0.001) and macular holes (*p* = 0.008) increased significantly with age. Cystoid macular edema was significantly associated with poorer visual acuity in RP patients with clear lens (*p* = 0.002).

**Conclusion:**

Cataracts and macular abnormalities are common in RP patients. Of the macular abnormalities, cystoid macular edema may have a negative effect on BCVA in RP patients with clear lens. Therefore, optical coherence tomography screening in RP patients is highly recommended for early detection and treatment of maculopathy.

**Supplementary Information:**

The online version contains supplementary material available at 10.1186/s12886-020-01797-z.

## Background

Retinitis pigmentosa (RP) is the most common type of inherited retinal dystrophy, causing progressive degeneration of the retinal pigment epithelium (RPE) and photoreceptors [1]. RP prevalence is approximately 1/4000 and more than 1.5 million patients are affected worldwide [2]. Nyctalopia and blurred vision are the most common RP symptoms, but other rare symptoms (e.g. photophobia, metamorphopsia) also prompt RP patients to visit a doctor [1–3]. However, little systematic information has been published on the clinical symptoms that RP patients experience before diagnosis.

Ocular abnormalities, such as glaucoma, cataracts, maculopathy, etc., may occur as RP progresses [4]. The typical histopathological change in RP is thinning of the photoreceptors’ outer segments, which worsens as RP progresses [4, 5]. Although central vision acuity can remain normal for several years, anatomical macular abnormalities may occur in early-stage RP [6, 7]. The most common macular abnormalities in RP patients are epiretinal membrane (ERM) and cystoid macular edema (CME), which are detected by optical coherence tomography (OCT). Other macular abnormalities also accompanied by, such as macular holes (MH), vitreomacular traction syndrome (VMT) and choroid neovascularisation membrane (CNVM) [5–9]. To the authors’ knowledge, visual acuity and prevalence of ocular abnormalities have not been reported previously in a large cohort of RP patients in Western China.

Therefore, this study assessed ocular abnormalities in a large cohort of RP patients in Western China and investigated correlations between visual acuity and macular abnormalities.

## Methods

### Study design and subjects recruitment

The authors retrospectively extracted medical records of patients diagnosed with RP between January 2014 and January 2019 at Southwest Hospital/ Southwest Eye Hospital, Third Military Medical University (Army Medical University), Chongqing, China. The records included information on each patient’s age, sex, medical and surgical history, family history, complaints, best corrected visual acuity (BCVA), intraocular pressure, lens status and the slit-lamp anterior segment and dilated fundus examination from the first clinical presentation. RP diagnosis was based on: (1) presence of night blindness or blurred vision and peripheral visual field restriction; (2) characteristic fundus changes, such as pale optic disc, attenuated vessels and bone-spicule-like pigmentation deposits in the mid- or far-periphery; and (3) reduced or non-detectable full-field electroretinogram (ff-ERG) rod and cone amplitudes [1, 4, 5]. The exclusion criteria were: (1) a history of trauma; (2) a history of vitreoretinal surgery and intravitreal therapy; (3) pathological myopia; (4) other vascular retinopathies, such as hypertensive retinopathy, diabetic retinopathy, retinal periphlebitis, etc.; (5)age-related macular degeneration; (6) atypical RP, such as unilateral pigmentary retinopathy or sectorial pigmentary retinopathy; (7) secondary retinal pigmentosa; and (8) severe systemic diseases. The study was performed according to the Declaration of Helsinki and approved by the Ethics and Research Committee of Southwest Hospital, Army Medical University (KY2020096).

### Age of onset and functional examination

The age of onset (that is, of symptoms) was defined as the patient’s age subtracted from the year with a positive disease history. BCVA was measured with a Tumbling E chart and converted into the logarithm of the minimum angle of resolution (logMAR) value for analysis [10]. BCVA was classified according to the World Health Organization’s (WHO) category of vision as follows [2]: BCVA worse than 3/60 in the better eye was classified as blindness; BCVA of 3/60–6/18 in the better eye was classified as low vision; and BCVA of 6/18 or more was classified as normal. The researchers did not classify visual acuity according to the visual field. Full-field electroretinogram testing was performed according to the International Society of Clinical Electrophysiology of Vision’s standards [11].

### Imaging examination

The lens condition was classified as clear, cataract, pseudophakic or aphakic. A specialist diagnosed glaucoma based on the presence of glaucomatous optic neuropathy and intraocular pressure over 21 mmHg, with or without the presence of iridotrabecular contact [12]. The macular microstructure was examined with Spectral Domain OCT (SD-OCT) (Cirrus HD-OCT, Carl Zeiss Meditec, Dublin California, USA) or Heidelberg Spectralis OCT (Heidelberg Engineering, Heidelberg, Germany). Two experienced ophthalmologists independently evaluated the images. If the results differed, a third ophthalmologist re-evaluated them. The macular abnormalities were documented as: ERM, CME, MH (including lamellar and full-thickness MH), VMT and CNVM according to the following definitions [1, 13, 14]:

ERM was diagnosed with the presence of as avascular, fibrocellular membrane on the inner surface of the retina, often resulting from proliferative changes at the vitro-retinal interface.

CME was defined as the presence of cystoid spaces, appearing like small hypo-reflective lacunae with well-defined boundaries on two or more consecutive views of the radial scan in the macular area.

Lamellar-thickness macular hole (LMH) was defined as partial thickness defects of the macular area, with an irregular foveal contour and a schisis between the inner and the outer retinal layers and without any photoreceptor layer defects. Full-thickness macular hole (FTMH) was defined as a vertical split in the neurosensory layers of the foveal region.

VMT was characterized by a vitromacular adhesion that involved the foveal region from posterior hyaloid face, causing traction and distortion of the central macular.

CNVM was defined as cystic macular edema associated with a disruption of the Bruch membrane/retinal pigment epithelium (RPE) complex, accompanied by an avascular structure emanating from the deep capillary plexus and appearing as a hyperreflective lesion connected with the subretinal pigment epithelium. Other diseases that cause macular CNVM were excluded [9, 14].

### Molecular diagnosis and inheritance pattern

Some patients voluntarily underwent molecular diagnosis, and inheritance patterns were categorised according to the genetic test reports: autosomic dominant (AD), autosomic recessive (AR), X-linked (X-L) or sporadic (i.e., patients with negative genetic reports or no evidence of other affected family members). RP patients’ ages were divided into four groups for statistical analysis (≤15 years, 16–44 years, 45–64 years and ≥ 65 years).

### Statistical analyses

SPSS 22.0 was used to conduct analyses. Continuous variables, such as counselling age, age of onset and BCVA (logMAR), were expressed as means±standard deviation (SD) and were compared with independent sample t-tests. Categorical variables (sex, complaints, inheritance pattern, age group, lens condition and macular abnormalities) were presented as counts and percentages and compared with Chi-squared or Fisher’s exact tests. Multiple linear regression investigated the relationship between BCVA (logMAR) and macular abnormalities. Coefficients of the estimated regression (β), the corresponding statistical significance (*P*)*,* the exponential parameter and its confidence interval were presented for each factor. A *P*-value of < 0.05 was considered statistically significant.

## Results

A total of 2127 eyes belong to 1065 patients (493 [46.3%] female and 572 [53.7%] male, respectively.) were investigated. Table 1 describes the patients’ demographic characteristics. The number of eyes did not match the number of patients because three eyeballs in three female patients had been enucleated due to glaucoma. The mean ± SD counselling age at the patient’s first eye hospital visit was 41.9 ± 15.7 years (range: 3 to 83 years; females: 43.4 ± 16.0; males: 40.6 ± 15.3; *p* = 0.000). The mean ± SD age of onset of symptoms for RP patients was 21.9 ± 19.2 years (females: 24.1 ± 19.7; males: 20.0 ± 19.6; *p* = 0.000). A total of 352 of 1065 patients (33.1%) had a molecular diagnosis, and the most common inheritance pattern was AR (57.7%), followed by sporadic (27.6%), AD (8.8%) and X-L (6.0%) (Table 1).

**Table 1:** Demographic characteristics of the 1065 RP patients in this study

	Female	Male	Total
**No. patients**	493(46.3%)	572(53.7%)	1065(100%)
**No. Eyes**	983^a^(45.2%)	1144(53.8%)	2127(100%)
**Counseling Age (yrs)**	43.4 ± 16.0	40.6 ± 15.3	41.9 ± 15.7
**Mean age of onset (yrs)**	24.1 ± 19.7	20.0 ± 19.6	21.9 ± 19.2
**Inheritance pattern**			
**Autosomic dominant (No.)**	14(8.6%)	17(9.0%)	31(8.8%)
**Autosomic recessive (No.)**	99(60.7%)	104(55.0%)	203(57.7%)
**X-linked (No.)**	3(1.9%)	18(9.5%)	21(6.0%)
**Sporadic (No.)**	47(28.8%)	50(26.5%)	97(27.6%)
**Total (No.)**	163(100%)	189(100%)	352(100%)

^a^: Three eyeballs in three female patients were enucleated due to glaucoma

**Fig. 1:**
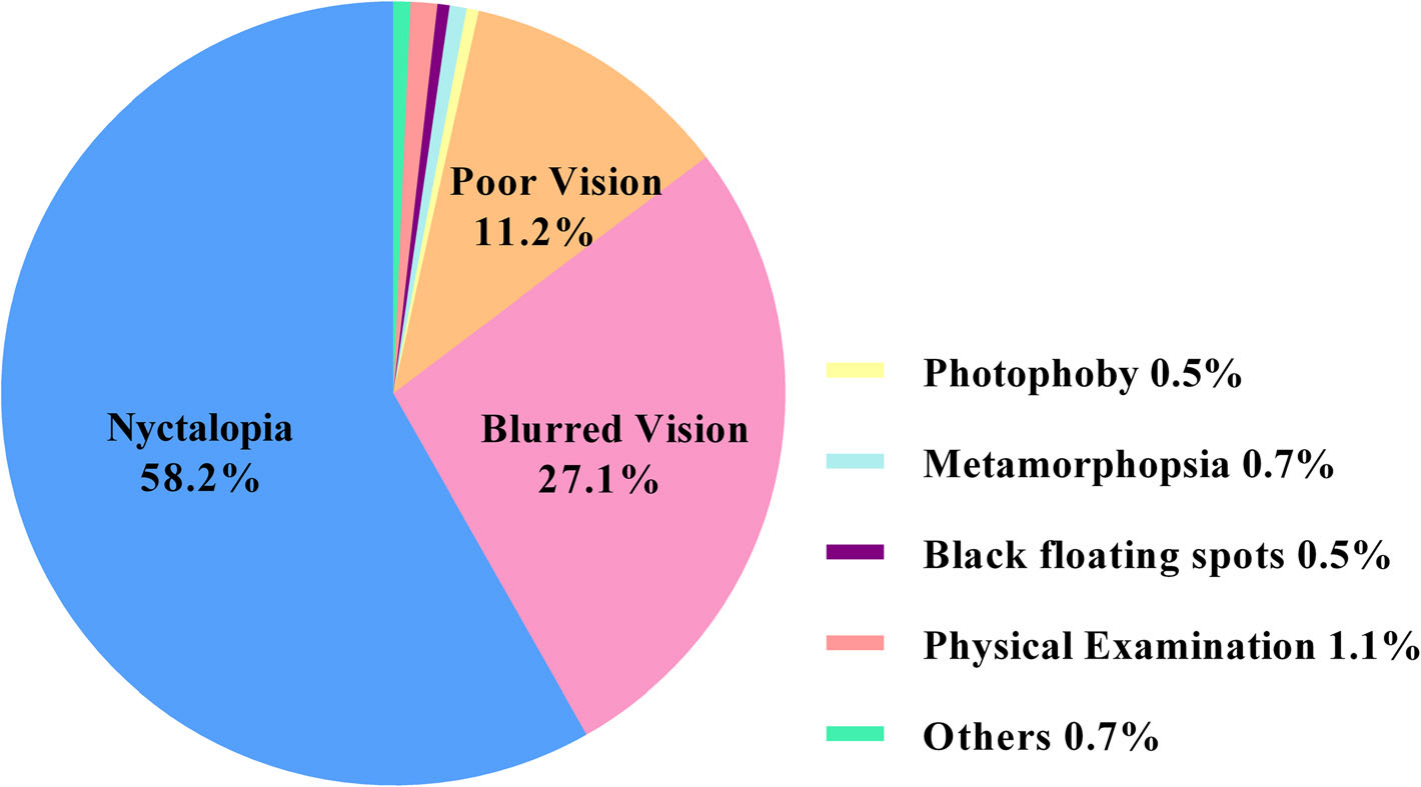
Distribution of chief complaints in the study cohort of patients with RP. The pie diagrams showed the sampled patients’ complaint were distributed as follows: nyctalopia (58.2%), blurred vision (27.1%), poor vision since childhood (≤15 years old, 11.2%), physical examination (1.1%), metamorphopsia (0.7%), photophoby (0.5%), black floating spots (0.5%) and other unusual symptoms (0.7%, including pain, photopsia, a narrow vision field and double vision)

Nyctalopia (58.2%) and blurred vision (27.1%) were the main visual complaints (Fig. 1). Among the patients’ in the study, 11.2% had experienced poor vision since childhood (≤15 years old). Other reasons for RP patients visiting the hospital included routine physical examination (1.1%), metamorphopsia (0.7%), photophoby (0.5%), black floating spots (0.5%) and other unusual symptoms (0.7%, including pain, photopsia, a narrow visual field and double vision).

**Fig. 2:**
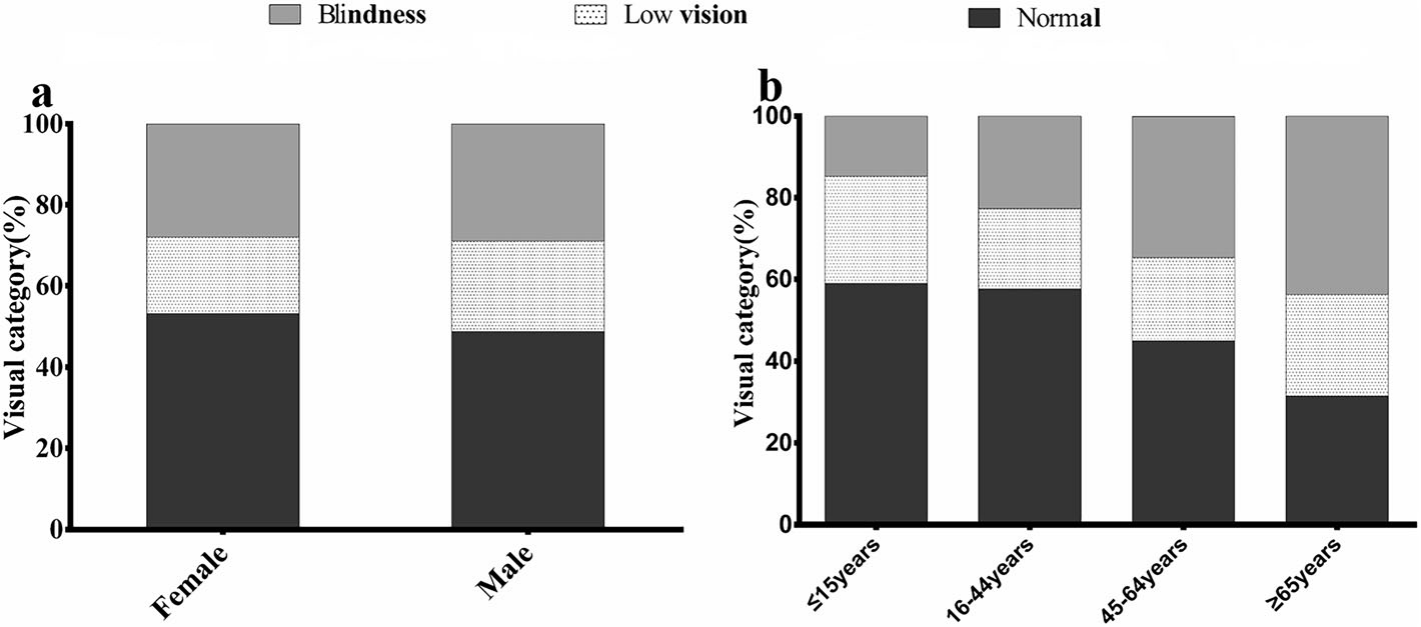
The best corrected visual acuity (BCVA) in the study cohort of RP patients, stratified by sex and age. Bar graph demonstrated the distribution of BCVA in the sampled RP patients at first clinical presentation. a: more than half of the RP patients presented visual acuity deterioration at first clinical presentation and no significant difference was observed between gender. b: patients over the age of 44 showed a lower proportion of normal vision than patients under 44 at first presentation, and the proportion of blindness at first presentation in patients over 44 (45–64y: 34.6%; ≥65y: 43.8%) was higher than in patients under 44 (≤15y: 8.5%; 16–44y: 14.8%). The percentage of blindness significantly increased with age in the sampled RP patients (*p* = 0.000)

The BCVA values in the better eye at first clinical presentation showed that 304 (28.5%) patients were blind, and 220 (20.7%) had low vision (Fig. 2a; Supplemental Table 1). Although the percentage of normal vision at first presentation in females (53.1%) was slightly higher than in males (48.8%), no significant sex difference was observed in visual acuity distribution (*p* = 0.283). Patients over the age of 44 showed a lower proportion (45–64y: 45%; ≥65y: 31.5%) of normal vision than patients under 44 (≤15y: 59.0%; 16–44y: 57.6%) at first presentation (Fig. 2b; Supplemental Table 2), and the proportion of blindness in patients over 44 years (45–64y: 34.6%; ≥65y: 43.8%) was higher than in patients under 44 years (≤15y: 8.5%; 16–44y: 14.8%). There was also a significant increase in the percentage of blindness with age (Fig. 2b; *p* = 0.000).

Cataracts were observed in 917 eyes (43.1%, 917/2127 eyes) of 469 patients in the sample (44.0%, 469/1065 patients). Pseudophakic and aphakic eyes were classed as presenting cataracts: of the 917 eyes in which cataracts were observed, pseudophakia was seen in 157 eyes (7.4%) of 95 patients (4.5%), and aphakia was seen in 22 eyes (1.0%) of 14 patients (0.7%). Glaucoma was found in 35 eyes (1.6%; 35/2127) of 21 patients (2.0%; 21/1065). Macular OCT was performed in 1388 eyes (65.3%; 1388/2127) of 704 patients (66.1%; 704/1065), and macular abnormalities were seen in 829 eyes (59.7%; 829/1388) of 481 patients (68.3%; 481/704).

**Fig. 3:**
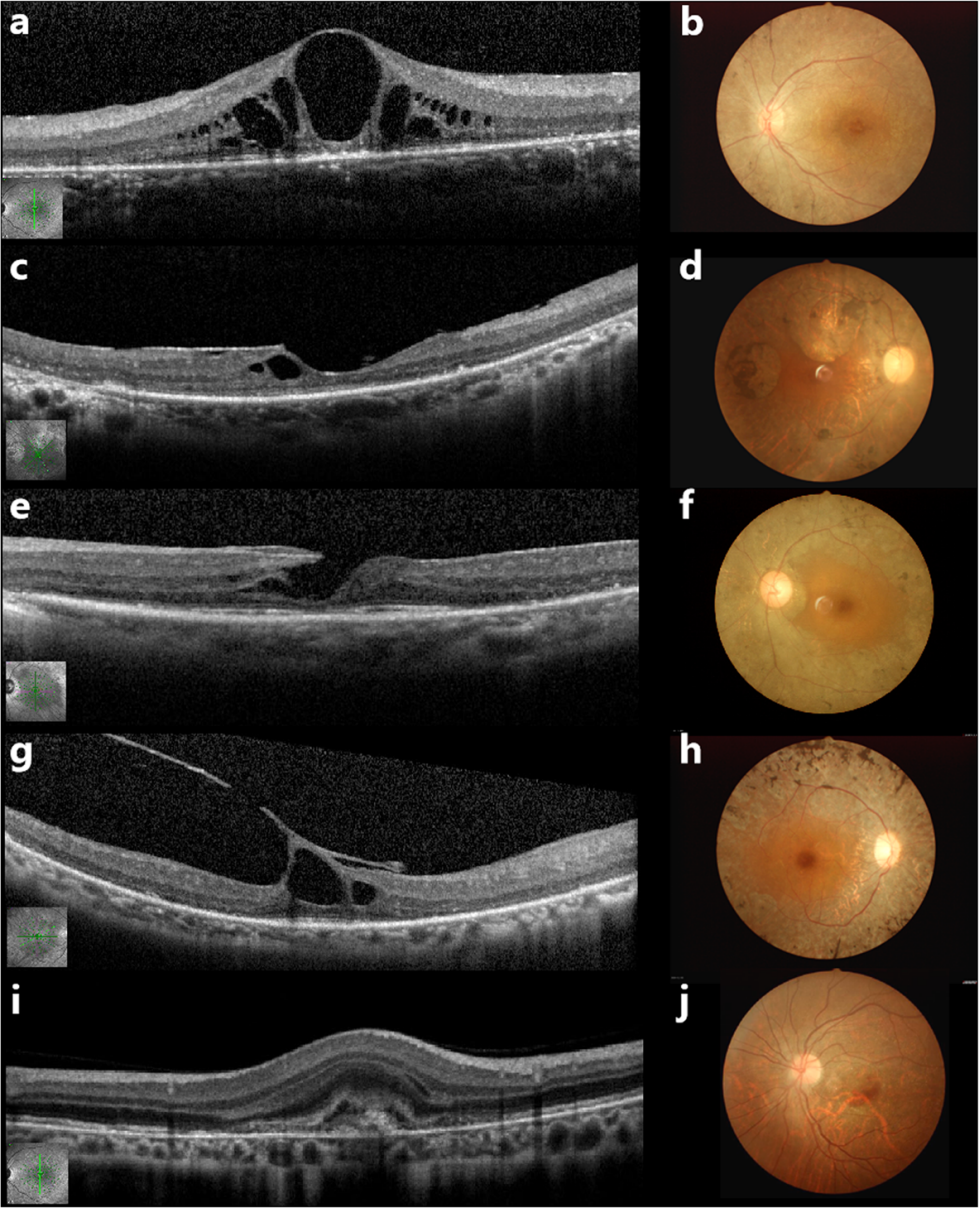
Representative optical coherence tomography (OCT) and corresponding fundus photography of RP patients with macular abnormalities. **a** An OCT image with cystic-appearing spaces in the left eye. **b** Fundus photograph of the left eye in picture a, showing bone spicule pigmentation in the mid-periphery and vessels attenuation. **c** OCT scan showed a homogenous layer of moderately reflective material, present on the inner retinal layer. **d** Fundus photography of the right eye in picture c, showed marked bone spicule pigmentation in the mid-periphery, waxy pallor of the optic disc and attenuated vessels. **e** OCT scan with lamellar macular hole. **f:** Fundus photography of the picture e, revealed bone spicule pigmentation in the mid-periphery and vessels attenuation. **g** OCT scan showed vitreomacular traction. **h** Fundus photography of the right eye in picture g, showed marked bone spicule pigmentation in the mid-periphery, waxy pallor of the optic disc and attenuated vessels. **i** OCT image showed disruption of the Bruch membrane/retinal pigment epithelium complex, accompanied by a hyper-reflective lesion connected with the subretinal pigment epithelium. **j** Fundus photography of the left eye in picture i, showed hemorrhage located in the inferior-temporal macular area

Typical OCT and corresponding fundus photography of macular abnormalities in RP patients are shown in Fig. 3 (a-j). The prevalence of macular abnormalities was as follows:

- ERM: 709 eyes (51.1%; 709/1388) of 418 patients (59.4%; 418/704).
- CME: 255 eyes (18.4%; 255/1388) of 150 patients (21.3%; 150/704).
- VMT: 33 eyes (2.4%; 33/1388) of 25 patients (3.6%; 25/704).
- MH: 32 (2.3%; 32/1388 eyes) of 26 patients (3.7%; 26/704).
- CNVM in one eye (0.05%; 1/,2127) of one female patient (0.09%; 1/1065).

**Fig. 4:**
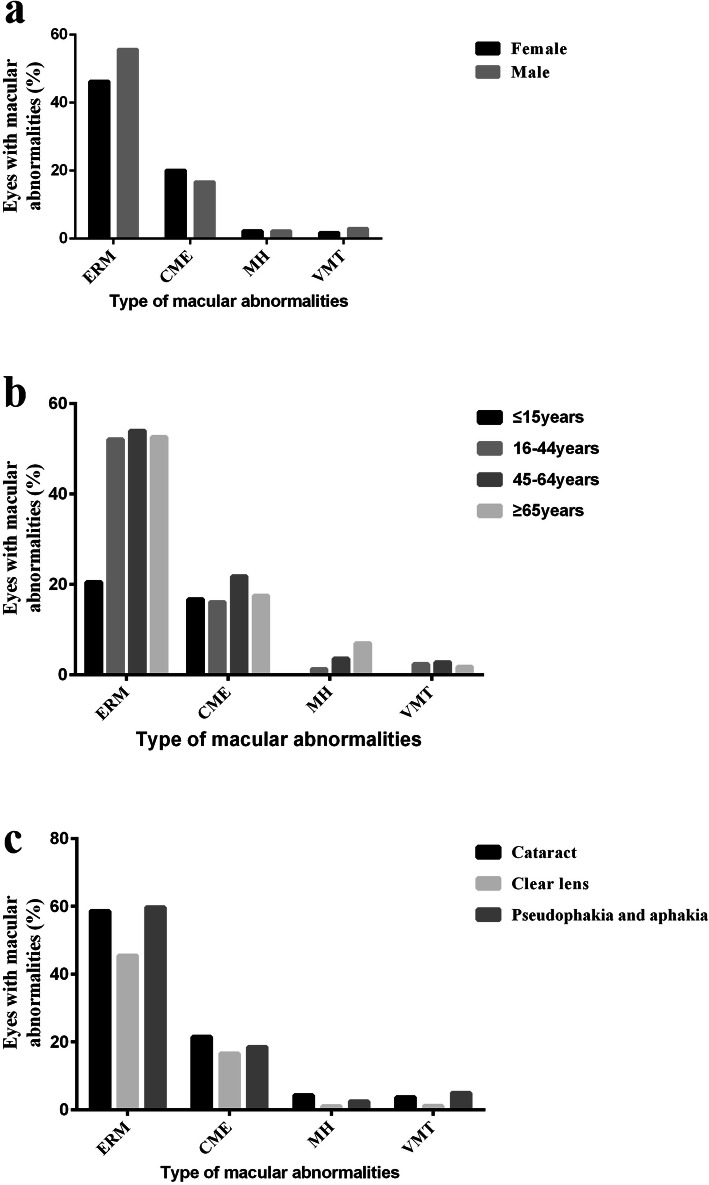
Classification of macular abnormalities in the study cohort of RP patients, stratified by (**a**) Sex; (**b**) Age; (**c**) Lens status. **a** Bar graph showed no significant differences among the classifications of macular abnormalities and sex (CME: *p* = 0.193; VMT: *p* = 0.176; MH: *p* = 0.383), except for ERM (males: 55.7%; females: 46.3%; *p* = 0.006). **b** This bar graph revealed prevalence of ERM (*p* = 0.001) and MH (*p* = 0.008) were significantly increased with age, while no differences were observed in CME (*p* = 0.283) and VMT (*p* = 0.619) distributions among age groups. **c** ERM (*p* < 0.001) and VMT (*p* = 0.003) were significantly more frequent in pseudophakic and aphakic eyes than in unoperated eyes (clear lens and cataracts). Abbreviations: ERM: epiretinal membranes; CME: cystoid macular edema; MH: macular hole; VMT: vitreomacular traction syndrome

Supplemental Tables 3, 4, 5 present the macular abnormality frequencies (stratifying patients according to sex, age and lens status) and show the corresponding statistical analysis. The results showed no significant differences among the classifications of macular abnormalities and sex (Fig. 4a, CME: *p* = 0.193; VMT: *p* = 0.176; MH: *p* = 0.383), except for ERM (males: 55.7%; females: 46.3%; *p* = 0.006).

MH and VMT were not found in any patients ≤15 years old. ERM (*p* = 0.001) and MH (*p* = 0.008) were significantly more prevalent in older RP patients, and prevalence increased with age (Fig. 4b; Supplemental Table 4). No differences were observed in the distribution of CME (*p* = 0.283) and VMT (*p* = 0.619) among the age group categories. Because some patients had undergone cataract surgery (pseudophakic and aphakic eyes), we compared macular abnormality distribution according to lens status. ERM (*p* < 0.001) and VMT (*p* = 0.003) were significantly more frequent in pseudophakic and aphakic eyes than in unoperated eyes (with clear lens and cataracts) (Fig. 4c; Supplemental Table 5). To eliminate the impact of cataracts on patients’ vision, we also analysed the relationship between macular abnormalities and BCVA (logMAR) for RP patients with clear lens, and poor BCVA seemed significantly associated with CME (*p* = 0.002) (Table 2).

**Table 2:** Linear regression between macular abnormalities and BCVA (logMAR) in RP patients with clear lens

Variable (eyes, no.)	***β***	***t***	***P*** value	Lower 95%CI	Upper 95%CI
**ERM (368)**	0.020	0.406	0.685	−0.076	0.115
**CME (134)**	−0.201	−3.058	0.002^*^	−0.329	−0.072
**MH (9)**	0.194	0.842	0.400	−0.258	0.646
**VMT (10)**	0.167	0.756	0.450	−0.266	0.599

*ERM* epiretinal membrane, *CME* cystoid macular edema, *MH* macular hole, *VMT* vitreomacular traction syndrome (*) = Significant values

## Discussion

To the best of our knowledge, this is the first study to report on the prevalence of ocular abnormalities in a large cohort of RP patients in Western China and to investigate the relationship between BCVA and macular abnormalities detected by OCT. The results showed that the most common ocular abnormalities were cataracts (43.1%) and macular abnormalities (59.7%). In patients with macular abnormalities, CME was significantly associated with poorer visual acuity in RP patients with clear lens.

Macular abnormalities distributed in our study as follows: ERM (51.1%), CME (18.4%), VMT (2.4%), MH (2.3%), and CNVM (0.05%). Although the prevalence of macular abnormalities in people of different ages and ethnicities and in regions around the world varies, the frequency of ERM, VMT and MH in the general population has been reported as follows: 9.1% [15], 1.6–2.4% [16] and 1.6‰-2.7‰ [17], respectively. While the distribution of CME and CNVM varies according to age and disease [16, 18]. ERM has been reported as the second most frequent macular abnormality in RP patients (Table 3). Testa et al’s retrospective study of the prevalence of macular abnormalities in Usher syndrome patients [13] and reported a prevalence of 47%. The authors also found the most frequent abnormalities were ERM (19% of eyes), followed by CME (15.7%), VMT (14.2%), and MH (3.0%) [13]. However, the prevalence of ERM in our study was much higher (51.1%) than in previous studies [4, 21, 22], which may be due to the application of spectral domain-OCT with higher resolution, different genetic backgrounds, and different diagnostic methods. We noted the presence of ERM when even a subtle, hyperreflective lesion adhered to the inner retinal surface, regardless of other abnormalities being present. The mechanisms of ERM formation remain unclear. However, it may include idiopathic preretinal glial cell proliferation, inflammation revealed by an elevated aqueous flare, and chronic macular-vitreous traction [19, 22–24].

**Table 3:** Comparison of the prevalence of ocular abnormalities in RP patients with previous studies

First author	Coun-try	Subjects/ Eyes (No.)	Macular abnormalities (Eyes / %)					Cataract (Eyes/%)	Glaucoma (Eyes/%)
			Total	CME	ERM	VMT	MH		
**Hajali** [6]	USA	124/248		115/46.4					
**Testa** [1]	Italy	581/1161	524/45.1	237/20.4	181/15.6	58/5.0	23/2.0		
**Fujiwar-a** [19]	Japan	117/206	73/35.4		73/35.4				
**Liew** [5]	UK	169/338		172/50.9	77/22.8				
**Testa** [13]	Italy	134/268	126/47.0	42/15.7	51/19.0	38/14.2	8/3.0		
**Lee** [20]	Korea	365/365						175/47.9	
**Onakpo-ya OH** [2]	Nigeria	96/192	70/36.5					38/20	22/11.5
**Our study**	China	1065/2127	829/59.7	255/18.4	709/51.1	33/2.4	32/2.3	917/43.1	35/1.6

*CME* Cystoid macular edema, *ERM* Epiretinal membrane, *VMT* Vitreomacular traction syndrome, *MH* Macular hole

In our study, CME was the second most common macular abnormality, which is inconsistent with the results from an Italian population in which Testa et al. investigated macular abnormalities in 581 RP subjects [1], finding that the most frequent abnormality was CME (20.4% eyes), followed by ERM (15.6%), VMT (5%), and MH (2%). CME varies from 5.5 to 49% in RP patients [4, 21]. The exact mechanism of CME in RP remains unclear; however, it may include the breakdown of the blood-retinal barrier secondary to the degeneration of the RPE and/or the Müller cells, anti-retinal antibodies and traction from ERM and VMT. There is no consensus on the relationship between CME and visual acuity in RP patients [22, 23] . Sandberg et al. found that retinal thinning (due to cell loss) and retinal thickening (due to presumed edema) appeared to be significantly associated with lower visual acuity in RP patients [25]. Yoshida et al. demonstrated that a normal preoperative ellipsoid zone (EZ), also called the inner/outer segment junction (IS/OS), was significantly related to better BCVA in RP patients [26]. Because cataracts and posterior subcapsular cataract (PSCs) were prevalent in RP subjects and were negatively correlated with BCVA, we analysed the relationship between macular abnormalities and BCVA (logMAR) only in eyes with a clear lens. CME appeared to be significantly associated with poor BCVA in our study. The exact relationship between maculopathy and visual acuity requires greater attention in future studies.

CNVM are rare, and until recently, no data have shown the prevalence of CNVM in RP patients. For several years, this information could only be obtained through case reports [9, 14, 27]. In our study, CNVM was observed in only one eye of one female patient (prevalence: approximately 0.09%). It has been proposed that photoreceptor cell degeneration and choriocapillaris damage may lead to the formation of CNVM [14].

Although the exact pathophysiology of maculopathy secondary to RP is not fully understood, various pharmacological and surgical treatments for macular abnormalities have been reported [28]. Topical carbonic anhydrase inhibitors (CAI) [29], grid laser photocoagulation [30], intravitreal therapy with corticosteroids or anti-vascular endothelial growth factor (VEGF) agents [9, 31], and pars plana vitrectomy [32] may be effective for early treatment.

The prevalence of cataract in different age, ethnicities and regions around the world varies. Hashemi et al. conducted a systematic review and meta-analysis of cataract prevalence and found that the age-standardized pooled prevalence estimate (ASPPE) of cataract in population-based was 17.20% [33]. Cataracts were the second most common ocular abnormality in our RP patients, and lens opacity developed at a relatively younger age than in the general population. The prevalence (43.1%) in our study was similar to the 47.9% prevalence reported by Lee et al. among Korean patients (Table 3) [20]. PSCs are the most typical morphological abnormalities and occur in 63–83.9% of RP patients [21, 34, 35]. However, lens status was determined through medical records, and cataract type was unidentifiable in our study. Glaucoma is another ocular abnormality that is prevalent among RP subjects. There is some evidence to suggest similar genetic backgrounds for glaucoma and RP [12, 36]. Ko et al. reported 3.64-fold greater odds of developing acute angle closure in patients with RP than in the general population [37]. In our study, the prevalence of glaucoma was 2%, which was much lower than the 11.5% reported by Onakpoya et al. [2] and the 7.5% reported by Eballe et al. [38], although similar to the prevalence in the general population (2–3%) [12]. The reason for the lower rate may be due to our larger sample cohort and our study’s retrospective nature.

More than half the RP patients in our study presented with visual acuity deterioration at their first clinical presentation, and the proportion of blindness and low vision as defined by the BCVA were 28.5 and 20.7%, respectively. We defined low vision or blindness according to central visual acuity and did not consider visual field defects. The rates of low vision and blindness in the RP subjects were actually much higher than these results show.

This study had the advantage of having a large sample size, and it assessed various ocular abnormality distributions and visual acuity simultaneously. However, it had several limitations: it was retrospective, and other ocular abnormalities and details, such as corneal nebulae, cataract and glaucoma types, remained unexplored. In addition, most patients had no molecular diagnosis, and we could not sufficiently investigate ocular abnormalities in different genetic subtypes. Further studies, including prospective investigations and studies with more patients with genetic diagnoses, are needed to explore the relationship between the course of RP and BCVA and /or to clarify the relationship between the genetic phenotypes of RP and BCVA.

## Conclusion

The results revealed that ocular abnormalities associated with RP are varied and have high prevalence, especially cataracts and macular abnormalities. Additionally, severe visual impairment was prevalent at the first clinical presentation of RP patients at a single eye hospital in Western China. For macular abnormalities, CME may negatively affect BCVA in RP patients with eyes with clear lens. It is essential to evaluate the macular structure with OCT when accessing RP patients’ visual function.

## Supplementary Information

**Additional file 1: Supplemental Table 1.** The BCVA in the study cohort of patients with retinitis pigmentosa stratifying by sex

**Additional file 2: Supplemental Table 2.** The BCVA in the study cohort of patients with retinitis pigmentosa stratifying by age

**Additional file 3: Supplemental Table 3.** Classification of macular abnormalities in the study cohort of patients with retinitis pigmentosa stratifying by sex

**Additional file 4: Supplemental Table 4.** Classification of macular abnormalities in the study cohort of patients with retinitis pigmentosa stratifying by age

**Additional file 5: Supplemental Table 5.** Classification of macular abnormalities in the study cohort of patients with retinitis pigmentosa stratifying by lens status

## Data Availability

Data could be available from the corresponding author by reasonable inquire.

## Abbreviations

AD: Autosomic dominant
ASPPE: Age-standardized pooled prevalence estimate
AR: Autosomic recessive
BCVA: Best corrected visual acuity
CAI: Carbonic anhydrase inhibitor
CME: Cystoid macular edema
CNVMs: Choroid neovascularisation membranes
ERMs: Epiretinal membranes
EZ: Ellipsoid zone
ff-ERG: Full-field electroretinogram
IS/OS: Inner/outer segment junction
ISCEV: International Society of Clinical Electrophysiology of Vision
MH: Macular hole
OCT: Optical coherence tomography
PSC: Posterior subcapsular cataract
RP: Retinitis pigmentosa
RPE: Retinal pigment epithelium
VEGF: Vascular endothelial growth factor
VMT: Vitreomacular traction syndrome
