# Supplementary material for: Ocular abnormalities in a large patient cohort with retinitis pigmentosa in Western China
Source: BMC Ophthalmol. 2021 Jan 18;21:43. doi: 10.1186/s12886-020-01797-z (PMC7812647; doi:10.1186/s12886-020-01797-z)
Supplement: Supplementary file 1 — Additional file 1: Supplemental Table 1. The BCVA in the study cohort of patients with retinitis pigmentosa stratifying by sex [file 12886_2020_1797_MOESM1_ESM.pdf]

Supplemental Table 1 The BCVA in the study cohort of patients with retinitis pigmentosa stratifying by sex

	Overall (n=1065)	Female (n=493)	Male (n=572)	χ^2	*P* value
**Normal**	541 (50.8%)	262 (53.1%)	279 (48.8%)		
**Low vision**	220 (20.7%)	93 (18.9%)	127 (22.2%)	2.521	0.283
**Blindness**	304 (28.5%)	138 (28.0%)	166 (29%.0)		
