# Supplementary material for: Ocular abnormalities in a large patient cohort with retinitis pigmentosa in Western China
Source: BMC Ophthalmol. 2021 Jan 18;21:43. doi: 10.1186/s12886-020-01797-z (PMC7812647; doi:10.1186/s12886-020-01797-z)
Supplement: Supplementary file 2 — Additional file 2: Supplemental Table 2. The BCVA in the study cohort of patients with retinitis pigmentosa stratifying by age [file 12886_2020_1797_MOESM2_ESM.pdf]

**Supplemental Table 2** The BCVA in the study cohort of patients with retinitis pigmentosa stratifying by age

| | Overall (n=1065) | ≤15years (n=61) | 16-44years (n=489) | 45-64years (n=433) | ≥65years (n=73) | $\chi^2$ | P value |
|---|---|---|---|---|---|---|---|
| **Normal** | 541 (50.8%) | 36 (59.0%) | 287 (57.6%) | 195 (45.0%) | 23 (31.5%) | | |
| **Low vision** | 220 (20.7%) | 16 (26.2%) | 98 (19.7%) | 88 (20.3%) | 18 (24.7%) | 36.956 | <0.001 |
| **Blindness** | 304 (28.5%) | 9 (14.8%) | 113 (22.7%) | 150 (34.6%) | 32 (43.8%) | | |
