# Supplementary material for: Ocular abnormalities in a large patient cohort with retinitis pigmentosa in Western China
Source: BMC Ophthalmol. 2021 Jan 18;21:43. doi: 10.1186/s12886-020-01797-z (PMC7812647; doi:10.1186/s12886-020-01797-z)
Supplement: Supplementary file 3 — Additional file 3: Supplemental Table 3. Classification of macular abnormalities in the study cohort of patients with retinitis pigmentosa stratifying by sex [file 12886_2020_1797_MOESM3_ESM.pdf]

Supplemental Table 3 Classification of macular abnormalities in the study cohort of patients with retinitis pigmentosa stratifying by sex

	**Overall**		**Male**		**Female**			
	Eyes (n=1388)	Patients (n=704)	Eyes (n=706)	Patients (n=357)	Eyes (n=682)	Patients (n=347)	χ^2	P value
**ERM**	709(51.1%)	418(59.4%)	393 (55.7%)	230 (64.4%)	316 (46.3%)	188 (54.2%)	7.660	0.006*
**CME**	255(18.4%)	150 (21.3%)	118 (16.7%)	69 (19.3%)	137 (20.1%)	81 (23.3%)	1.692	0.193
**MH**	32 (2.3%)	26 (3.7%)	16 (2.3%)	11 (3.1%)	16 (2.3%)	15 (4.3%)	0.763	0.383
**VMT**	33(2.4%)	25 (3.6%)	21 (3.0%)	16 (4.5%)	12 (1.8%)	9 (2.6%)	1.832	0.176

ERM epiretinal membrane, CME cystoid macular oedema, MH macular hole, VMT vitreomacular traction syndrome

(*)=Significant values
