# Supplementary material for: Ocular abnormalities in a large patient cohort with retinitis pigmentosa in Western China
Source: BMC Ophthalmol. 2021 Jan 18;21:43. doi: 10.1186/s12886-020-01797-z (PMC7812647; doi:10.1186/s12886-020-01797-z)
Supplement: Supplementary file 4 — Additional file 4: Supplemental Table 4. Classification of macular abnormalities in the study cohort of patients with retinitis pigmentosa stratifying by age [file 12886_2020_1797_MOESM4_ESM.pdf]

Supplemental Table 4 Classification of macular abnormalities in the study cohort of patients with retinitis pigmentosa stratifying by age

	Overall		≤15years		16-44years		45-64years		≥65years		χ^2	P value
	Eyes (n=1388)	Patients (n=704)	Eyes (n=78)	Patients (n=39)	Eyes (n=720)	Patients (n=364)	Eyes (n=533)	Patients (n=271)	Eyes (n=57)	Patients (n=30)		
**ERM**	709 (51.1%)	418 (59.4%)	16 (20.5%)	11 (28.2%)	375 (52.1%)	220 (60.4%)	288 (54.0%)	170 (62.7%)	30 (52.6%)	17 (56.7%)	17.236	0.001*
**CME**	255 (18.4%)	150 (21.3%)	13 (16.7%)	7 (17.9%)	116 (16.1%)	69 (19%)	116 (21.8%)	68 (25.1%)	10 (17.5%)	6 (20.0%)	3.809	0.283
**MH**	32 (2.3%)	26 (3.7%)	0 (0%)	0 (0%)	9 (1.3%)	7 (1.9%)	19 (3.6%)	16 (5.9%)	4 (7.0%)	3 (10.0%)	10.950	0.008*
**VMT**	33 (2.4%)	25 (3.6%)	0 (0%)	0 (0%)	17 (2.4%)	12 (3.3%)	15 (2.8%)	12 (4.4%)	1 (1.8%)	1 (3.3%)	1.595	0.619

ERM epiretinal membrane, CME cystoid macular oedema, MH macular hole, VMT vitreomacular traction syndrome

(*)=Significant values Note: MH and VMT were tested by Fisher exact method
