# Supplementary material for: Ocular abnormalities in a large patient cohort with retinitis pigmentosa in Western China
Source: BMC Ophthalmol. 2021 Jan 18;21:43. doi: 10.1186/s12886-020-01797-z (PMC7812647; doi:10.1186/s12886-020-01797-z)
Supplement: Supplementary file 5 — Additional file 5: Supplemental Table 5. Classification of macular abnormalities in the study cohort of patients with retinitis pigmentosa stratifying by lens status [file 12886_2020_1797_MOESM5_ESM.pdf]

Supplemental Table 5 Classification of macular abnormalities in the study cohort of patients with retinitis pigmentosa stratifying by lens status

	**Overall (n=1388)**	**Cataract (n=461)**	**Clear Lens (n=808)**	**Pseudophakic and aphakia (n=119)**	χ^2	**P value**
**ERM**	709 (51.1%)	270 (58.6%)	368 (45.5%)	71 (59.7%)	23.762	<0.001*
**CME**	255 (18.4%)	99 (21.5%)	134 (16.6%)	22 (18.5%)	4.683	0.096
**MH**	32 (2.3%)	20 (4.3%)	9 (1.1%)	3 (2.5%)	13.577	0.001*
**VMT**	33 (2.4%)	17 (3.7%)	10 (1.2%)	6 (5.0%)	11.573	0.003*

ERM epiretinal membrane, CME cystoid macular oedema, MH macular hole, VMT vitreomacular traction syndrome

(*)=Significant values
